# New insights into lysosomal ferroptosis-related prognostic signatures in prostate cancer: evidence from bulk and single-cell transcriptomic analysis

**DOI:** 10.3389/fcell.2026.1861663

**Published:** 2026-08-04

**Authors:** Xudong Zhu, Xixi Ji, Hao Liu, Haoran Chen, Jiazheng Wang

**Affiliations:** 1 Department of Oncology, Guang’anmen Hospital, China Academy of Chinese Medical Sciences, Beijing, China; 2 Department of Pediatrics, Beijing Hospital of Traditional Chinese Medicine, Beijing, China; 3 Department of Oncology, Affiliated Traditional Chinese Medicine Hospital of Guangzhou Medical University, Guangzhou, China

**Keywords:** FTH1, lysosomal ferroptosis, prognostic risk model, prostate adenocarcinoma, tumor immune microenvironment

## Abstract

**Background:**

Prostate adenocarcinoma (PRAD) is a leading reason of cancer-related death in men worldwide, yet reliable biomarkers for accurate risk stratification are lacking. This study sought to build and test a lysosomal ferroptosis-related prognostic risk model for PRAD.

**Methods:**

This study merged single-cell RNA sequencing (scRNA-seq) and bulk RNA sequencing datasets. Differentially expressed genes (DEGs) from the TCGA-PRAD cohort were intersected with 39 lysosomal ferroptosis-related genes (LFRGs). Univariate Cox regression and random survival forest (RSF) algorithms were applied to build a prognostic risk model, which was validated in two separate cohorts (GSE70768; GSE70769). Immune infiltration, drug sensitivity, and single-cell transcriptomic analyses were subsequently performed. Finally, the expression and potential mechanism of hub genes were investigated in experimental samples.

**Results:**

Seven candidate genes were identified, from which MMD and FTH1 were chosen to create a prognostic risk model. The model achieved AUC values of 0.90, 0.89, and 0.87 at 1-, 2-, and 3-year timepoints in TCGA-PRAD, with consistent performance across both validation cohorts. High-risk patients displayed an immunosuppressive microenvironment noted for enhanced myeloid-derived suppressor cells, regulatory T cells, upregulation of 23 immune checkpoint genes, higher TIDE scores, and increased tumor mutational burden (TMB). Drug sensitivity analysis identified differential responses to 3 agents after FDR correction. Single-cell analysis revealed myeloid-predominant expression of MMD and FTH1, with divergent pseudotime kinetics and enhanced KRAS, IL2-STAT5, and mTORC1 signaling, with MIF–CD74 as a key intercellular communication axis. The hub genes were validated in experimental samples and these findings suggest a potential therapeutic strategy combining FTH1 degradation with PD-L1 blockade, although the immunological mechanisms require further validation in immunocompetent models.

**Conclusion:**

This study presented a novel and potentially useful lysosomal ferroptosis-related prognostic risk model that effectively stratified PRAD patients by survival outcome and therapeutic response, providing a valuable framework for personalized clinical decision-making.

## 1 Introduction

Prostate adenocarcinoma (PRAD) is the second common malignancy among men worldwide, with adenocarcinoma being the most prevalent histological type (≥99%) (Raychaudhuri et al., 2025). Due to its indolent progression and susceptibility to curative interventions, low-grade prostate cancer is linked to a high overall survival rate. As the disease advances clinically, its management becomes increasingly complex (Karwacki et al., 2022). Current primary treatment modalities for PRAD include radical surgery, radiotherapy, androgen deprivation therapy (ADT), and chemotherapy. However, the immunosuppressive tumor microenvironment (TME) renders prostate cancer a challenging target for most immunotherapies (Zarrabi et al., 2019). Therefore, the identification of new prognostic biomarkers and therapeutic targets is of significant clinical importance.

Ferroptosis is a unique form of regulated cell death propelled by the accumulation of iron-dependent lipid peroxidation within cells, distinct from apoptosis, necrosis, and autophagy in terms of morphology, biochemical characteristics, and genetic regulation. Recent studies have indicated that ferroptosis represents a form of immunogenic cell death (ICD), which augments the capable of innate immune cells to detect tumor cells (Chen et al., 2021). Consequently, ferroptosis can effectively promote the conversion of “cold” tumors into “hot” tumors, ultimately improving cancer patients’ response to immunotherapy (Tang et al., 2020; Gao et al., 2022; Hänggi and Ruffell, 2023). Furthermore, depletion of serine hydroxymethyltransferase 2 (SHMT2) disrupts one-carbon metabolism, elevates reactive oxygen species (ROS) levels, and reduces ATP levels, subsequently activating autophagy and promoting autophagosome–lysosome fusion. This process leads to lysosomal membrane permeabilization (LMP) and ultimately induces cell death (Liu et al., 2023). Lysosomes contain substantial amounts of iron and is essential for iron homeostasis by aggregating signals from various subcellular locations and cell death pathways (Sumneang et al., 2020; Rizzollo et al., 2021). The accumulation of iron within lysosomes and its subsequent transfer to mitochondria have been identified as key events mediating ferroptosis (Li et al., 2024). Studies have demonstrated that the ferroptosis inhibitor Liproxstatin–1 exerts cytoprotective acts by deactivating iron within lysosomes, while the ferroptosis inducer RSL3 can initiate lipid oxidation on the lysosomal membrane, further underscoring the central role of lysosomes in the initiation of ferroptosis (Cañeque et al., 2025). However, research on the overall expression profiles, prognostic value, and functional roles of lysosomal ferroptosis-associated genes across distinct cellular subpopulations within the prostate cancer TME remains scarce.

This study aims to integrate bulk and single-cell transcriptomic data, focusing on the expression signatures of lysosomal ferroptosis-associated genes, to systematically screen for genes significantly linked to the prognosis of PRAD patients and to probe the potential regulatory mechanisms of these genes. Then, the specific expression patterns of these prognostic genes within distinct cell populations of the TME will be analyzed at single-cell sharpness. This study is projected to offer a deeper understanding of the role of lysosomal ferroptosis in prostate cancer progression, offer novel molecular genes for prognostic assessment.

## 2 Materials and methods

### 2.1 Data acquisition

On 19 August 2025, transcriptomic data for PRAD tumor specimens and controls were sourced from The Cancer Genome Atlas (TCGA) database. It included data from the TCGA-PRAD cohort, with a total of 483 tumor and 51 normal control specimens, with overall survival information available for 408 tumor samples. Additionally, two independent validation cohorts were soured from the Gene Expression Omnibus (GEO) database. The GSE70768 dataset (platform GPL10558) contained transcriptomic profiles from 111 tumor samples of PRAD patients. The GSE70769 dataset (platform GPL10558) contained transcriptomic profiles from 92 tumor specimens of PRAD patients. Furthermore, the scRNA-seq dataset GSE153892 (platform GPL18573) was acquired from the GEO, encompassing transcriptomic profiles from 3 PRAD tumor tissues and 3 normal control specimens. A sum of 39 LFRGs were soured from the literature (Cañeque et al., 2025) (Supplementary Table S1).

### 2.2 Identification of candidate genes (CGs) and functional characteristics

Differential expression analysis was performed to determine dysregulated genes in PRAD via the “DESeq2” package (v 1.40.2) (Love et al., 2014). Transcriptomic data from PRAD tumor samples and controls within the TCGA-PRAD cohort were analyzed, and DEGs were defined (|log_2_FC| > 0.5, adj p < 0.05). To identify lysosomal ferroptosis-associated genes in PRAD, an intersection analysis was executed with the “ggvenn” package (v 0.1.10) (https://CRAN.R-project.org/package=ggvenn). To elucidate their biological roles, gene set enrichment analysis (GSEA) was performed in the TCGA-PRAD cohort. Reference gene set representing pathways was obtained from the Molecular Signatures Database (MSigDB). Specifically, the “c2.cp.kegg_legacy.v2025.1.Hs.symbols” was utilized. For each candidate gene, Spearman correlation coefficients (cor) between each gene and all other genes were measured via the “psych” package (v 2.2.9) (https://CRAN.R-project.org/package=psych). Genes were then ranked based on their cor values in descending order to generate a ranked gene list for GSEA input. Subsequent GSEA was conducted via the “clusterProfiler” package (v 4.10.1) (Wu et al., 2021) (|NES| > 1, p < 0.05).

### 2.3 Prognostic genes screening and model construction

Univariate Cox regression analysis was implemented on the CGs by transcriptomic data from the TCGA-PRAD specimens with available survival information (n = 408). This analysis was executed via the “survival” package (v 3.8-3) (Lei et al., 2023), with genes displaying a hazard ratio (HR) ≠ 1 and a p < 0.2 deemed statistically marked (Soo-Jin et al., 2013). The finds were depicted via the “forestplot” package (v 2.0.1) (Liao et al., 2022; Li-Fei et al., 2025). The proportional hazards (PH) assumption for these significant genes was assessed, with a p value >0.05 indicating no significant violation of the assumption. Subsequently, a RSF model was built based on the genes to measure the risk score for each patient.

### 2.4 Prognostic risk model assessment and validation

First, the optimum cutoff value was determined to classify patients into high- and low-risk groups (HRG and LRG). The distribution of patient risk scores (ordered ascending) and the corresponding survival status and survival time were illustrated. Subsequently, Kaplan-Meier (K-M) survival traces were yielded via the “survminer” package (v 0.5.0) (Liu et al., 2021), and the log-rank test was applied for evaluation (p < 0.05). To measure the predictive accuracy and discriminatory power of the prognostic risk model at 1-, 2-, and 3-year time points, time-dependent receiver operating characteristic (ROC) curve analysis was executed via the “survivalROC” package (v 1.0.3.1) (Heagerty et al., 2000) (AUC > 0.6). Next, the prognostic risk model was further validated by the aforementioned methods. Then, clinical data for 335 PRAD patients were sourced from the TCGA-PRAD cohort, and Kruskal–Wallis and Wilcoxon tests were adopted to contrast differences in risk scores among different clinical subgroups (age, T stage, N stag, M stage) (p < 0.05).

### 2.5 GSEA

Differential gene expression analysis comparing the HRG versus the LRG was first implemented via the “DESeq2” package on the TCGA-PRAD cohort. Genes were subsequently ranked based on their log2FC values, sorted from largest to smallest. GSEA was then employed to recognize pathways enriched at the extremes of this ranked gene list. Reference gene set representing pathways was obtained from the MSigDB, specifically, the “c2.cp.kegg_legacy.v2025.1. Hs.symbols” was utilized. GSEA was then performed using the “clusterProfiler” package to determine enriched biological pathways (p < 0.05, |NES| > 1). Furthermore, to elucidate the biological functions associated with individual prognostic genes in PRAD, GSEA was performed in the TCGA-PRAD cohort samples with available survival information (n = 408) (|NES| > 1, p < 0.05). Use the same gene set as reference data.

### 2.6 Immune cell infiltration analysis and evaluation of TME

Immune infiltration analysis was executed by the ssGSEA technique to assess the proportion of 28 predefined immune cell types via the “GSVA” package (v1.46.0) (Hänzelmann et al., 2013). The Wilcoxon test was adopted to compare the infiltration levels of each immune cell type between the two cohorts (p < 0.05). To investigate correlations between markedly differentially infiltrated immune cells, and between the prognostic genes and these immune cells, Spearman correlation coefficient values were calculated via the “psych” package (p < 0.05, |cor| > 0.3). Next, comprehensively evaluate the TME using an evaluation algorithm. This analysis was performed on all 408 PRAD samples from the TCGA-PRAD cohort with available survival information. The ESTIMATE method was adopted to quantify the abundance of stromal cells and immune cells within tumor tissues, yielding three distinct scores: the stromal, the immune, and the combined ESTIMATE score. Differences in the distribution of stromal, immune, and ESTIMATE scores between the two cohorts were statistically appraised by the Wilcoxon test (p < 0.05).

### 2.7 Immune checkpoint gene expression and immunotherapy response prediction

The expression levels of 38 immune checkpoint genes were compared between the HRG and LRG in the TCGA-PRAD cohort (n = 408, samples with survival information). The 38 immune checkpoint genes were obtained from a previously published study (Zhang X. et al., 2022). Differences in each immune checkpoint gene between the two risk cohorts were measured via the Wilcoxon test (p < 0.05). To further evaluate the potential response to immune checkpoint blockade (ICB) therapy, the tumor immune dysfunction and exclusion (TIDE) score was calculated for each patient via the TIDE web platform. Lower TIDE scores indicate a higher likelihood of responding to ICB therapy. Differences in TIDE scores between two cohorts were contrasted via the Wilcoxon test (p < 0.05). Additionally, Spearman correlation analysis was performed to examine the relationship between risk scores and TIDE scores (|cor| > 0.3, p < 0.05).

### 2.8 Somatic mutation analysis and drug sensitivity prediction

Somatic mutation data for the TCGA-PRAD cohort were acquired. TMB, as the count of somatic mutations per megabase, was calculated for each specimen. To characterize the mutational landscape of HRG and LRG, waterfall plots were yielded to illustrate the top 20 most commonly mutated genes. Drug sensitivity analysis was conducted to measure potential differences in therapeutic response between the risk groups. Drug response data were soured from the Genomics of Drug Sensitivity in Cancer (GDSC) database. The half-maximal inhibitory concentration (IC_50_) values for these drugs were predicted using the R package “pRRophetic” (v 0.5) (Geeleher et al., 2014). To reduce false-positive findings caused by multiple comparisons, P values derived from Wilcoxon rank-sum tests were adjusted using the Benjamini–Hochberg false discovery rate (FDR) method. Statistical significance was defined as adjusted P value (FDR) < 0.05. Only drugs meeting this threshold were considered for downstream analysis. Correlation analysis between prognostic genes and significantly differential drugs was performed using Spearman correlation (|cor| > 0.3, p < 0.05).

### 2.9 The scRNA-seq data processing and cell type labeling

The scRNA-seq data from tumor specimens and controls were prepared via the “Seurat” package (v 5.1.0) (Hao et al., 2024). To assure data quality and robustness, rigorous quality control (QC) filtering was applied to retain cells and genes meeting the following criteria: (1) the count of unique molecular identifiers (UMIs) per cell (nCount_RNA) spanned from 500 to 7,000; (2) the count of assayed genes per cell (nFeature_RNA) ranged from 200 to 2,000; and (3) the fraction of mitochondrial gene expression was below 10%. After QC filtering, data from different specimens were integrated via the “IntegrateData” function. Next, the filtered data were standardized via the “NormalizeData” function. Subsequently, the top 2,000 highly variable genes (HVGs) were determined via the FindVariableFeatures function. Principal component analysis (PCA) was executed on the scaled data of these HVGs to reduce dimensionality. Principal components (PCs) that recorded the majority of biological variation were selected for subsequent dimensionality reduction and cell clustering. Uniform manifold approximation and projection (UMAP) was implemented for non-linear dimensionality reduction and visualization. Cell clustering was conducted by the FindNeighbors and FindClusters functions within the “Seurat” package, employing the previously identified PCs and defining the resolution parameter to 0.4. Finally, cell type annotation for the identified clusters was executed rely on the expression patterns of canonical marker genes curated from established literature (Huang et al., 2023).

### 2.10 Single-cell expression profiling of prognostic genes and pseudotime trajectory analysis

Differences in cell type proportions between PRAD specimens and controls were appraised. The expression distribution of these genes was examined by all identified cell types. Cell types showing enriched prognostic genes were recognized as key cell types for in-depth analysis. Key cell types were extracted and processed b dimensionality reduction. Subsequent sub-clustering of key cell types was executed, setting the resolution parameter to 0.1. To investigate the potential differentiation trajectory of key cell types, pseudotemporal ordering analysis was implemented with the “monocle” package (v 2.30.1) (Trapnell et al., 2014). Cells were ordered along a reconstructed pseudotime trajectory rely on their gene expression profiles. Cells were also partitioned into distinct states according to their position along this pseudotime trajectory, facilitating visualization of the distribution of different key cell type subclusters across inferred developmental stages. The expression dynamics of prognostic genes along the reconstructed pseudotime trajectory were analyzed.

### 2.11 Functional profiling of key cell types and inference of cell-cell communication networks

To explore biological functional differences of the key cell types between PRAD samples and controls, functional enrichment analysis was performed. Gene set variation analysis (GSVA) was applied via the “GSVA” package (v 1.46.0). The Hallmark gene set collection (file: h.all.v2024.1.Hs.symbols.gmt) was downloaded. GSVA assigned pathway activity scores to individual cells, enabling the exploration of enrichment for specific biological pathways within cell types. To infer transcription factor regulatory activity, the DoRothEA algorithm was applied. Metabolic pathway activity within annotated cell types was quantified using the R package “scMetabolism” (v 0.2.1) (Wang et al., 2023). In addition, ligand-receptor interactions mediating cell-cell communication were postulated via the “CellChat” package (v 1.6.1) (Jin et al., 2021).

### 2.12 Quantitative real-time PCR (qPCR)

First, total RNA was extracted from the specimens via TRIzol reagent, and cDNA was synthesized via reverse transcription. qPCR reactions were executed on a LightCycler 480 (Roche) using Platinum SYBR Green Master Mix (catalog no. 11,744,500, Yingjie Life Sciences). The relative mRNA expression levels of MMD and FTH1 were calculated using the 2^(−ΔΔCt)^ method, with GAPDH as the internal control. The primer are shown in Table 1.

**TABLE 1:** Polymerase chain reaction primer sequence.

Names	Application	Sequences (5’→3′)
Primers		
MMD forward	qPCR	CCG​CTA​CAA​GCC​AAC​TTG​CTA​T
MMD reverse	qPCR	AGT​CCC​ATT​CCA​TAA​ATC​CAT​G
FTH1 forward	qPCR	CCC​CCA​TTT​GTG​TGA​CTT​CAT
FTH1 reverse	qPCR	GCC​CGA​GGC​TTA​GCT​TTC​ATT

### 2.13 Western blot

Proteins were isolated by RIPA lysis buffer with protease inhibitors. Protein amounts were quantified by BCA assay (Thermo Fisher Scientific). Protein specimens (20–50 μg) were electrophoresed on SDS-PAGE, transferred to PVDF membranes, and blocked with 5% non-fat milk. Membranes were probed overnight at 4 °C with primary antibodies against MMD (20226, CST), FTH1 (3998, CST), GPX4 (ab125066, Abcam), and ACSL4 (ab155282, Abcam) (1:1,000), followed by secondary antibody (1:2,000) for 1 h at room temperature. Signals were depicted via the ChemiDoc XRS system (Bio-Rad) and examined with ImageJ, with normalization to β-actin.

### 2.14 Animals and treatments

Vital River (Beijing, China) supplied Humanized male NOD/SCID mice (6 weeks old, average weight 20 ± 3 g), to establish a C4-2 xenograft model. The humanized SCID model was selected to provide a partially reconstituted immune background for evaluating immune-checkpoint-related intervention in an otherwise immunodeficient xenograft setting. Subcutaneous tumors were created bilaterally on the dorsal flank, and tumor volumes were quantified at least biweekly through digital calipers and calculated as (π/6) × (L × W^2^). Upon study completion, mice were sacrificed, and tumors were excised and weighed. Once tumours reached ∼50 mm^3^ in size, mice were assigned and categorized into distinct treatment cohorts: Vehicle control, Positive drug control group receiving 10  mg kg^−1^ enzalutamide by oral gavage 5 days per week for 3 weeks; for the test group, mice were intraperitoneally administered 200 μg anti-CD274 monoclonal antibody (Biolegend, 329702) plus AY-4 (MCE, HY-174867) 10 mg/kg by oral gavage 5 days per week for 3 weeks. Anesthesia was induced in mice through the inhalation of isoflurane. During the induction phase, the isoflurane (Merck, 792632) concentration was maintained at 3%–5%, with an oxygen flow rate of 0.3–0.5 L/min. Following the loss of the righting reflex, the isoflurane concentration was reduced to 1%–3% for the maintenance of anesthesia. Throughout the anesthetic period, body temperature was sustained using a heating pad, and the depth of anesthesia was monitored by assessing respiration and the pedal withdrawal reflex. The experimental animals that were given euthanasia placed in a carbon dioxide anesthesia chamber (Yuyanbio, CL-1000-S3). The carbon dioxide gas valve was opened, The CO_2_ gas was delivered into the closed chamber at a flow rate of 10% of the chamber volume per minute (i.e., 50 mL/min for a 500 mL chamber). and after the animals gradually lost consciousness, the carbon dioxide concentration was increased to 100%. The animals exhibited a state of unconsciousness, characterized by the absence of a toe pinch reflex or loss of muscle tone. Ventilation was continued for 2 min to confirm death. In accordance with IACUC guidelines, tumor size was kept below 2.0 cm in any dimension across all treatment arms, and animals were promptly euthanized upon reaching this limit. Raw tumor volumes and/or weights from all studies are provided in the Source Data files. All Animal experimental protocols were authorized by the Animal Experiment Ethics Committee of Guang’anmen Hospital, China Academy of Chinese Medical Sciences.

### 2.15 Immunohistochemistry

Immunohistochemistry was executed implementing an EnVision Detection System (DAKO, Glostrup, Denmark). Tissue sections were affixed to glass slides, deparaffinized, rehydrated via a gradient of xylene and ethanol, and subjected to antigen retrieval by heating in a citrate buffer. After blocking with 5% BSA at 20 °C–25 °C to diminish nonspecific binding, the sections were cultured overnight with the specific primary antibody against Ki-67 (ab15580, Abcam), GPX4 antibodies (ab125066, Abcam) and ACSL4 antibodies (ab155282, Abcam) (1:200) at 4 °C. Thereafter, the sections were washed with PBS and treated with the secondary antibody at room temperature for 30 min. For color development, the sections were labeled with DAB for 1 min and 20 s and counterstained with hematoxylin for 2 min. After nuclear staining, dehydration, and air drying, the sections were mounted with Permount and coverslips, analyzed, and photographed under a light microscope (BX51, OLYMPUS).

### 2.16 Statistical analysis

Bioinformatics analyses were implemented with the R (v 4.2.2). Continuous variables were contrast via the Wilcoxon test. Survival analyses were implemented by K-M curves with log-rank tests. *p < 0.05 or adjusted p < 0.05.

## 3 Results

### 3.1 Recognition of CGs and functional characteristics

**FIGURE 1:**
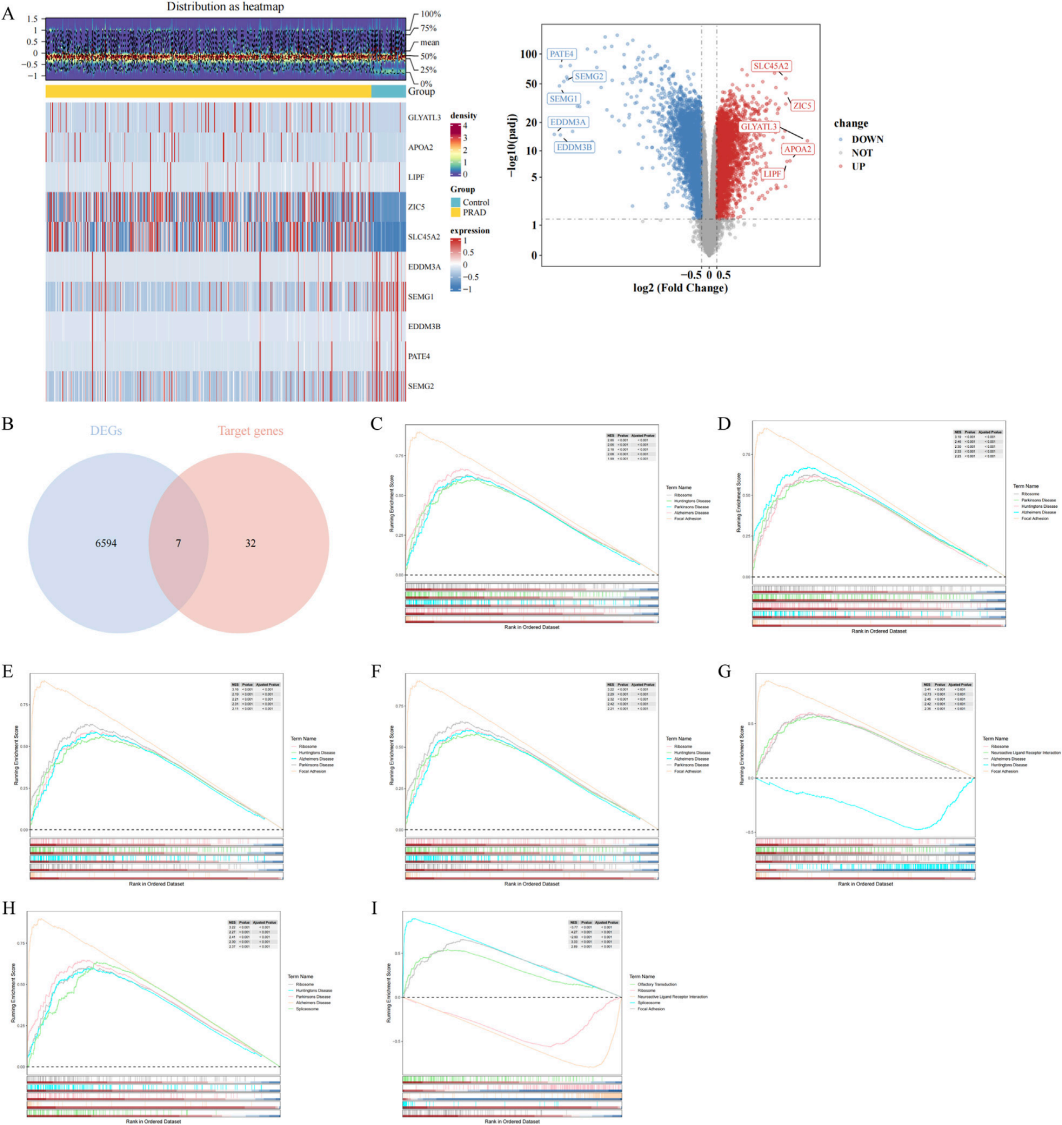
Identification of candidate genes and functional characterization in PRAD. **(A)** DEGs between PRAD tumor samples and controls in the TCGA-PRAD cohort (|log_2_FC| > 0.5, adjusted p < 0.05). **(B)** Venn diagram illustrating the intersection of DEGs with 39 predefined lysosomal ferroptosis-related genes, identifying seven candidate genes: CTSB, FTH1, TNFAIP3, SLC7A5, NEDD4, MMD, and MT3. **(C–I)** GSEA pathway enrichment profiles for individual candidate genes. Enrichment plots show significantly enriched KEGG pathways for **(C)** CTSB, **(D)** FTH1, **(E)** TNFAIP3, **(F)** SLC7A5, **(G)** NEDD4, **(H)** MMD, and **(I)** MT3 (|NES| > 1, p < 0.05).

Transcriptomic analysis of the TCGA-PRAD cohort revealed 6,601 DEGs between PRAD samples and controls, with 3,211 genes upregulated and 3,390 genes downregulated in tumors (|log_2_FC| > 0.5, adj p < 0.05) (Figure 1A; Supplementary Table S2). Intersection of these DEGs with 39 predefined LFRGs yielded seven CGs: CTSB, FTH1, TNFAIP3, SLC7A5, NEDD4, MMD, and MT3 (Figure 1B). Further, GSEA characterized the functional associations of the seven CGs within PRAD. All seven genes demonstrated consistent enrichment in core cancer-associated pathways, including ribosome, focal adhesion, spliceosome, oxidative phosphorylation, pathways in cancer, and endocytosis. Additionally, FTH1 and SLC7A5 exhibited specific enrichment in lysosome-related pathways, directly linking their expression to lysosomal iron homeostasis mechanisms. Several genes showed unique pathway associations: TNFAIP3 and MMD were enriched in regulation of actin cytoskeleton, NEDD4 demonstrated enrichment in neuroactive ligand-receptor interaction, and MT3 was distinctly enriched in olfactory transduction pathways, suggesting potential non-canonical functions in PRAD (Figures 1C–I; Supplementary Tables S3–S9). The convergent enrichment across fundamental cellular processes—including protein synthesis (ribosome), energy metabolism (oxidative phosphorylation), RNA processing (spliceosome), and membrane trafficking (endocytosis)—suggested these genes might coordinately regulate tumor cell growth and survival.

### 3.2 A prognostic risk model was built and demonstrated robust risk stratification capacity in PRAD

**FIGURE 2:**
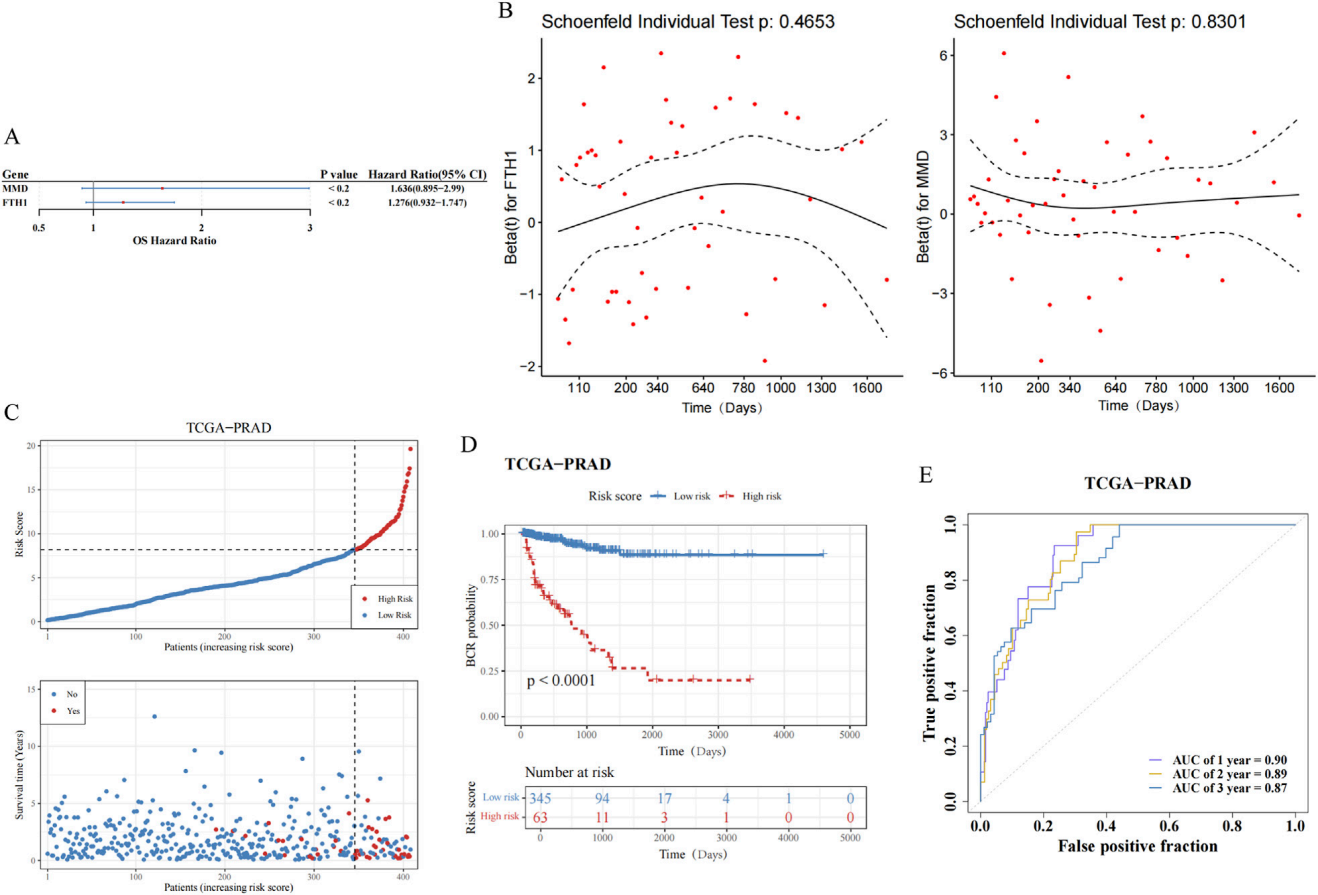
Construction and validation of a prognostic risk model in the TCGA-PRAD training cohort. **(A,B)** Univariate Cox regression analysis results. Forest plots present HRs with 95% confidence intervals for the seven candidate genes. All genes satisfied the PH assumption (Schoenfeld residual test, p > 0.05). **(C)** High-risk (n = 63) and low-risk (n = 345) patient groups stratified by the optimal risk score cut-off value (8.1867). **(D)** K-M survival curves comparing overall survival (OS) between high-risk and low-risk groups in the TCGA-PRAD cohort. **(E)** Time-dependent ROC curves for the prognostic risk model at 1-year, 2-year, and 3-year survival time points.

Univariate Cox regression analysis determined two genes markedly linked to overall survival in the TCGA-PRAD cohort (n = 408 samples with survival information), including MMD (HR = 1.636, 95% CI: 0.895–2.99) and FTH1 (HR = 1.276, 95% CI: 0.932–1.747) (p < 0.2). All genes satisfied the PH assumption (p > 0.05) (Figures 2A,B). A prognostic risk model rely on these genes was constructed using the RSF algorithm within the TCGA-PRAD cohort. Patients were stratified into HRG (n = 63) and LRG (n = 345) via the optimum risk score cut-off value (8.1867). Higher mortality rates were observed within the HRG (Figure 2C). K-M analysis proved markedly worse overall survival for HRG compared to LRG patients (p < 0.0001) (Figure 2D). The model demonstrated excellent predictive accuracy at 1-, 2-, and 3-year time points, with AUC values of 0.90, 0.89, and 0.87 (Figure 2E). These results established a prognostic risk model with strong predictive performance in PRAD, warranting further validation in separate cohorts.

### 3.3 External validation confirmed the prognostic utility of the prognostic risk model across independent cohorts

**FIGURE 3:**
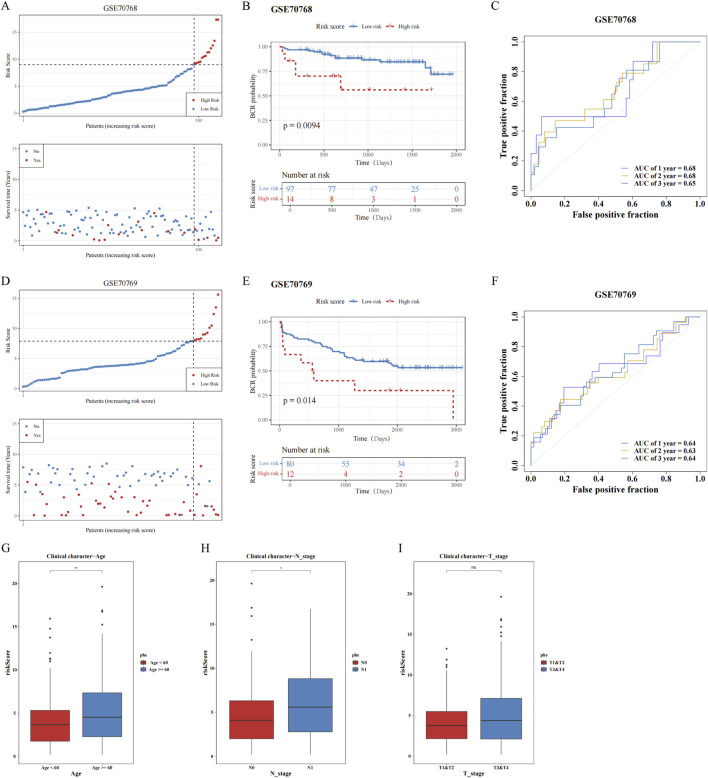
External validation of the prognostic risk model in independent GEO cohorts. **(A)** Stratification of patients in the GSE70768 cohort (n = 111) based on the model-derived risk score and optimal cut-off (8.9893), dividing patients into high-risk (n = 14) and low-risk (n = 97) groups, with mortality distribution displayed. **(B)** K-M survival curves for the GSE70768 cohort demonstrating that high-risk patients exhibited significantly worse overall survival (log-rank p = 0.0094). **(C)** Time-dependent ROC curves for 1-, 2-, and 3-year survival prediction in the GSE70768 cohort. **(D)** Stratification of patients in the GSE70769 cohort (n = 92) based on the model-derived risk score and optimal cut-off (7.869553), dividing patients into high-risk (n = 12) and low-risk (n = 80) groups, with mortality distribution displayed. **(E)** K-M survival curves for the GSE70769 cohort demonstrating that high-risk patients exhibited significantly worse overall survival (log-rank p = 0.014). **(F)** Time-dependent ROC curves for 1-, 2-, and 3-year survival prediction in the GSE70769 cohort. **(G–I)** Association between risk score and clinicopathological features. *p < 0.05; **p < 0.01.

The prognostic risk model was robustly validated in two independent external cohorts. In the GSE70768 cohort (n = 111), stratification using the model-derived risk score and its optimal cut-off (8.9893) yielded HRG (n = 14) and LRG (n = 97) (Figure 3A). Consistent with the TCGA-PRAD cohort, HRG patients exhibited significantly poorer survival (p = 0.0094) (Figure 3B). The AUC values for 1-, 2-, and 3-year prediction were 0.68, 0.68, and 0.65 (Figure 3C). Similarly, in the GSE70769 cohort (n = 92), application of the risk score model and its optimal cut-off (7.869553) divided patients into HRG (n = 12) and LRG (n = 80), with the HRG showing a higher proportion of deaths (Figure 3D). Survival analysis again revealed a significant disadvantage for the HRG (p < 0.014) (Figure 3E). The AUC values for this cohort were 0.64 (1-year), 0.63 (2-year), and 0.64 (3-year) (Figure 3F). Furthermore, marked associations were observed between the risk score and established clinical stage parameters (p < 0.05). The risk score displayed significant positive correlations with age (≥60 years vs. < 60 years, p = 0.032) and lymph node metastasis status (N1 vs. N0, p = 0.018), with higher risk scores observed in patients with adverse clinicopathological features (Figure 3G). These findings validated the prognostic capacity of the prognostic risk model in separate cohorts.

### 3.4 Functional enrichment analysis of risk groups and prognostic genes

**FIGURE 4:**
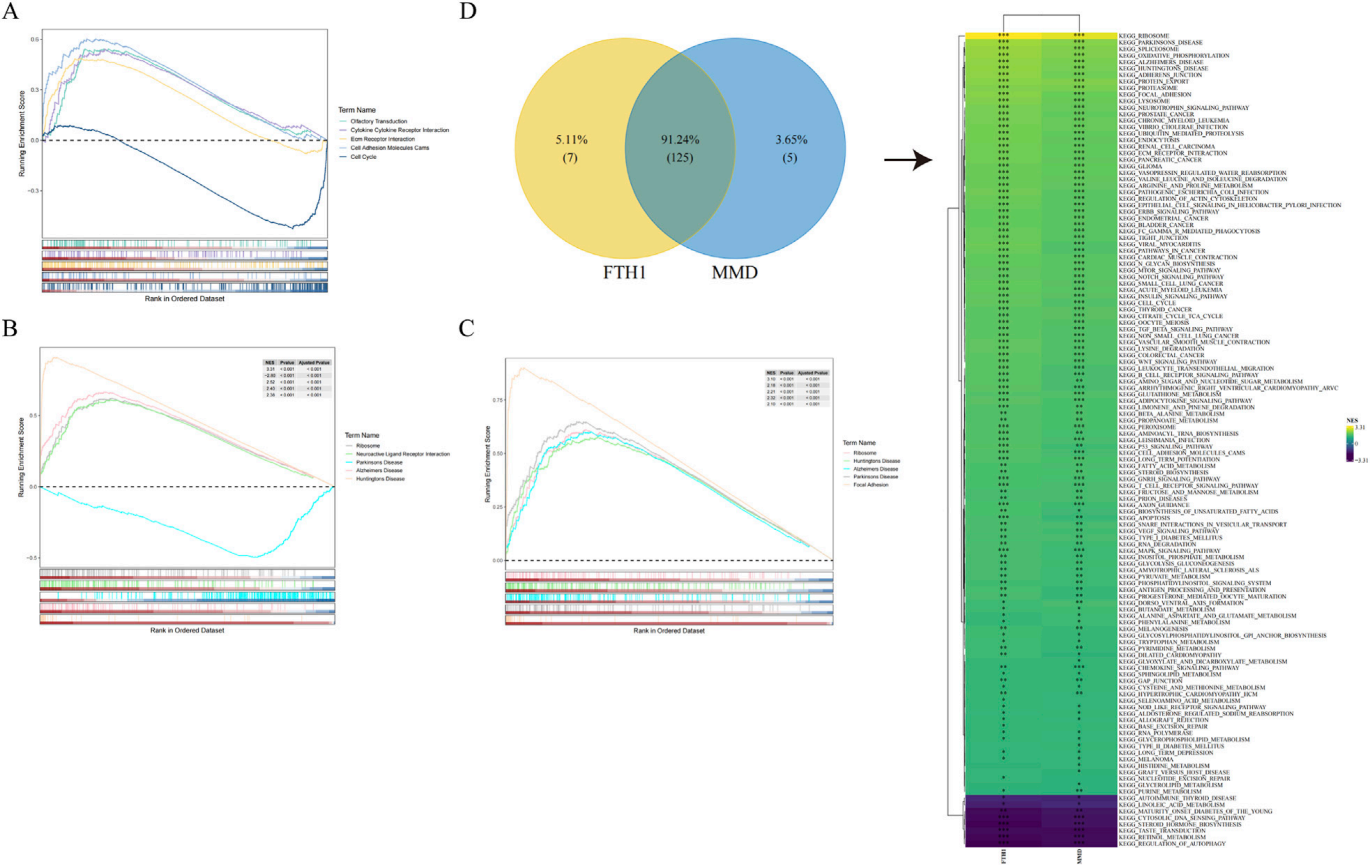
Functional enrichment analysis distinguished high-risk and low-risk PRAD phenotypes. **(A)** Significantly enriched KEGG pathways in high-risk versus low-risk PRAD patients identified by GSEA (|NES| > 1, p < 0.05). **(B)** Pathway enrichment landscape for MMD showing 130 significantly enriched KEGG pathways. **(C)** Pathway enrichment landscape for FTH1 showing 132 significantly enriched KEGG pathways. **(D)** Concordant pathway enrichment between MMD and FTH1 across major functional categories (|NES| > 1, p < 0.05).

GSEA revealed distinct biological pathway alterations between HRG and LRG PRAD patients. The genes exhibiting significant upregulation in the HRG were involved in immune-related pathways, including cytokine-cytokine receptor interaction, chemokine signaling pathway, cell adhesion molecules (CAMs), alongside pathways governing cell proliferation (cell cycle, ribosome) and tumor-stroma interactions (ECM-receptor interaction), etc. (|NES| > 1, p < 0.05) (Figure 4A; Supplementary Table S10). Further GSEA characterized the functional associations of the two prognostic genes within PRAD. MMD demonstrated significant enrichment in 130 KEGG pathways (Figure 4B; Supplementary Table S11), while FTH1 enriched in 132 pathways (Figure 4C; Supplementary Table S12), with 125 pathways (91.24%) overlapping between the two genes. Both genes showed concordant positive enrichment in core cellular processes essential for tumor growth, including ribosome, spliceosome, oxidative phosphorylation, adherens junction, protein export, proteasome, focal adhesion, and lysosome (NES >1, p < 0.05). Conversely, both genes exhibited negative enrichment in metabolic pathways, such as linoleic acid metabolism, and retinol metabolism, as well as in innate immune sensing pathways (cytosolic DNA-sensing pathway) and regulation of autophagy (NES < −1, p < 0.05) (Figure 4D). The convergent enrichment of MMD and FTH1 in anabolic pathways and suppression of catabolic/immune surveillance pathways suggested these genes coordinately reprogram cellular metabolism and immune evasion to sustain aggressive tumor phenotypes. Notably, the shared enrichment in lysosomal pathways reinforces the mechanistic link between lysosomal iron metabolism and PRAD progression, supporting the biological rationale for the prognostic risk model.

### 3.5 Immune microenvironment characterization

**FIGURE 5:**
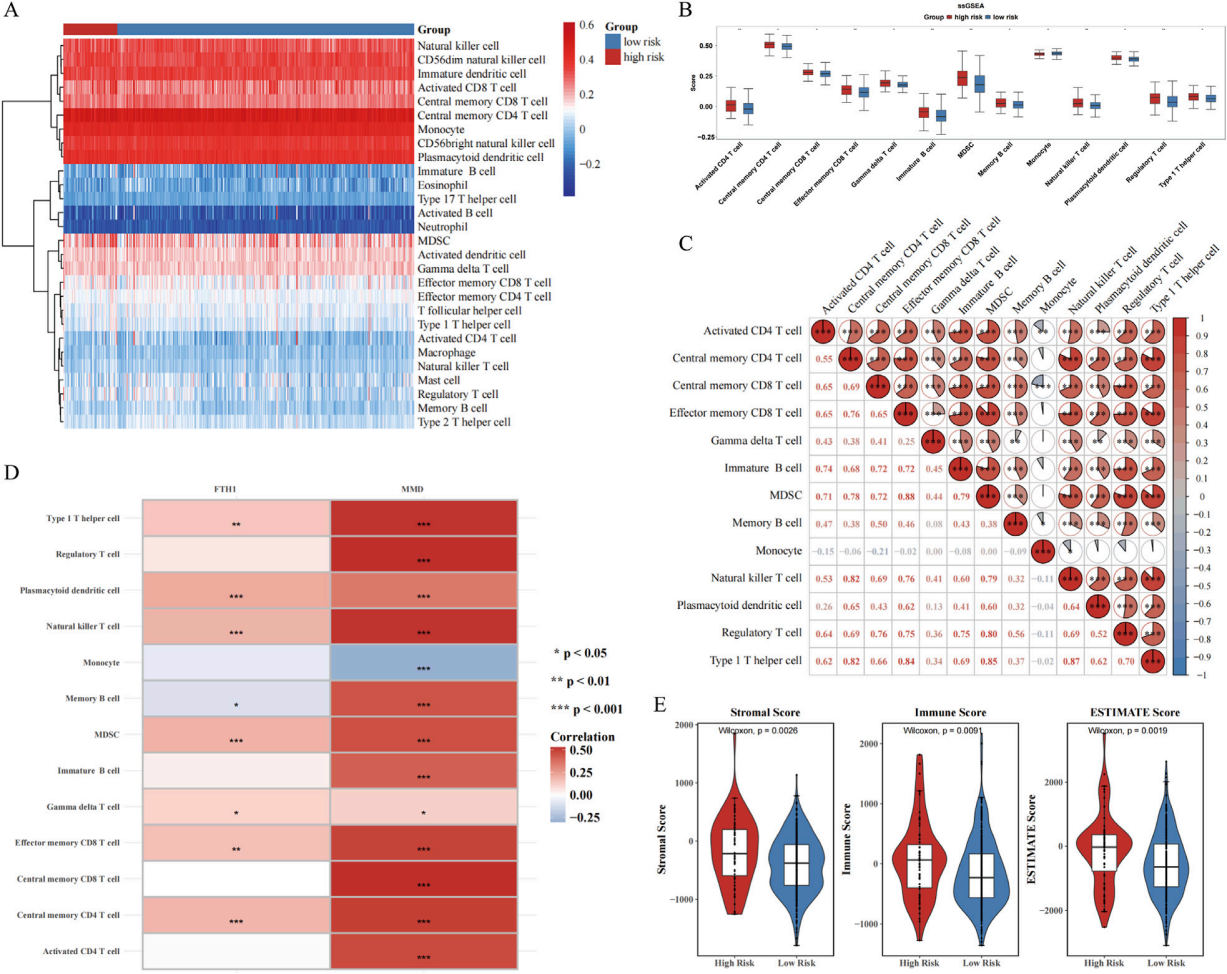
Characterization of tumor immune microenvironment in high-risk and low-risk PRAD. **(A,B)** The proportions of 22 immune cell types between high-risk and low-risk groups. **(C)** Correlation matrix (Spearman’s correlation) among immune cell populations. **(D)** Significant correlations between MMD expression and multiple immune cell populations (cor >0.5, p < 0.001). **(E)** Stromal scores, immune scores, and ESTIMATE scores between high-risk and low-risk groups.

The HRG demonstrated a markedly distinct immune infiltration profile contrasted to the LRG, characterized by significantly elevated proportions of immunosuppressive and regulatory cell populations, including activated CD4 T cells, effector memory CD8 T cells, myeloid-derived suppressor cells (MDSCs), natural killer T (NKT) cells, and regulatory T cells (Tregs) (all p < 0.05). Conversely, monocyte infiltration was markedly reduced in high-risk tumors (p < 0.05) (Figures 5A,B). Strong positive correlations were identified among most immune cells. Notable correlations included MDSCs and effector memory CD8 T cells (cor = 0.88, p < 0.001), NKT cells and central memory CD4 T cells (cor = 0.82, p < 0.001), and type 1 T helper cell with effector memory CD8 T cell (cor = 0.84, p < 0.001), MDSCs (cor = 0.85, p < 0.001), and NKT cells (cor = 0.87, p < 0.001) (Figure 5C). This co-infiltration pattern suggested coordinated activity within the tumor immune microenvironment. Specific associations were found between prognostic genes and immune cells. MMD expression showed significant positive correlations with type 1 T helper cells, Tregs, NKT cells, and central memory CD8 T cells (cor >0.5, p < 0.001) (Figure 5D). Furthermore, the ESTIMATE algorithm-based assessment displayed that HRG patients displayed markedly elevated stromal, immune, and ESTIMATE scores compared to the LRG (all p < 0.01) (Figure 5E), indicating increased abundance of both stromal and immune components in high-risk tumors.

### 3.6 High-risk PRAD exhibited elevated immune checkpoint expression, higher mutational burden, and distinct drug sensitivity profiles

**FIGURE 6:**
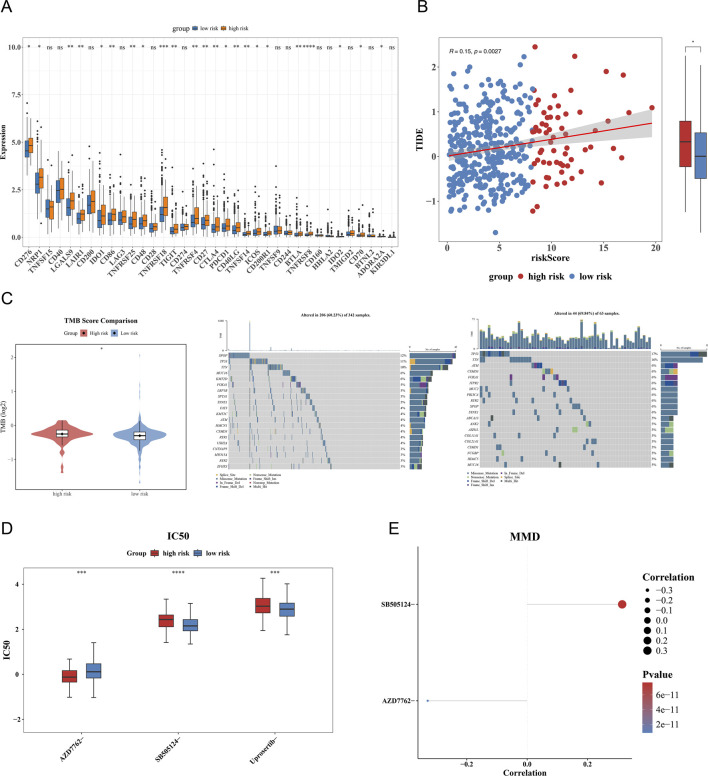
High-risk PRAD exhibited elevated immune checkpoint expression, elevated TMB, and distinct drug sensitivity profiles. **(A)** Expression levels of immune checkpoint genes between high-risk and low-risk groups. **(B)** Left panel: Scatter plot demonstrating positive correlation between risk score and TIDE score. Right panel: Box plot comparing tumor immune dysfunction and exclusion (TIDE) scores between risk groups (p < 0.05). **(C)** Somatic mutation profiles between risk groups. **(D)** Box plots of half-maximal inhibitory concentration (IC_50_) values for the top five differentially sensitive drugs between risk groups. **(E)** Significant correlations between prognostic gene expression and drug IC_50_ values.

Comparative analysis revealed 23 immune checkpoint genes exhibiting widespread upregulation in the HRG, with marked overexpression of LGALS9, LAIR1, CD86, TNFRSF25, TNFRSF18, BTLA, and TNFRSF8 (all p < 0.01) (Figure 6A). Furthermore, the TIDE score was markedly higher in the HRG and demonstrated a direct correlation with the risk score (p < 0.05) (Figure 6B). Distinct somatic mutation patterns were observed between HRG and LRG PRAD patients. Mutational profiling revealed distinct genomic alterations between risk groups. HRG patients demonstrated a markedly elevated TMB compared to the LRG (p < 0.05). In LRG, SPOP (12%), TP53 (11%), and TTN (10%) exhibited the highest mutation frequencies. HRG specimens showed elevated mutation frequencies in TP53 (17%) and TTN (16%) (Figure 6C). Based on the GDSC database and pRRophetic algorithm, IC50 values were estimated for candidate compounds in TCGA-PRAD samples. After multiple-testing correction using the Benjamini–Hochberg method, a total of three drugs showed significant differences in predicted IC50 between the high- and low-risk groups (FDR-adjusted p < 0.05) (Supplementary Table S13). Box plots illustrated the IC50 distributions of these three differential drugs (Figure 6D). These findings suggest that patients in different risk groups may exhibit distinct drug response profiles, indicating potential therapeutic heterogeneity in PRAD. Furthermore, correlation analysis based on these FDR-significant drugs revealed that MMD was significantly positively correlated with SB505124 (r = 0.31, p < 0.001) and significantly negatively correlated with AZD7762 (r = −0.33, p < 0.001) (Figure 6E), whereas FTH1 showed no significant correlation with any of the identified drugs. Collectively, this risk model not only predicts clinical outcomes but also reflects therapeutic response features, providing a reference framework for personalized treatment selection in PRAD.

### 3.7 Single-cell transcriptomic profiling indicated myeloid-specific expression of prognostic genes and developmental dynamics in PRAD

**FIGURE 7:**
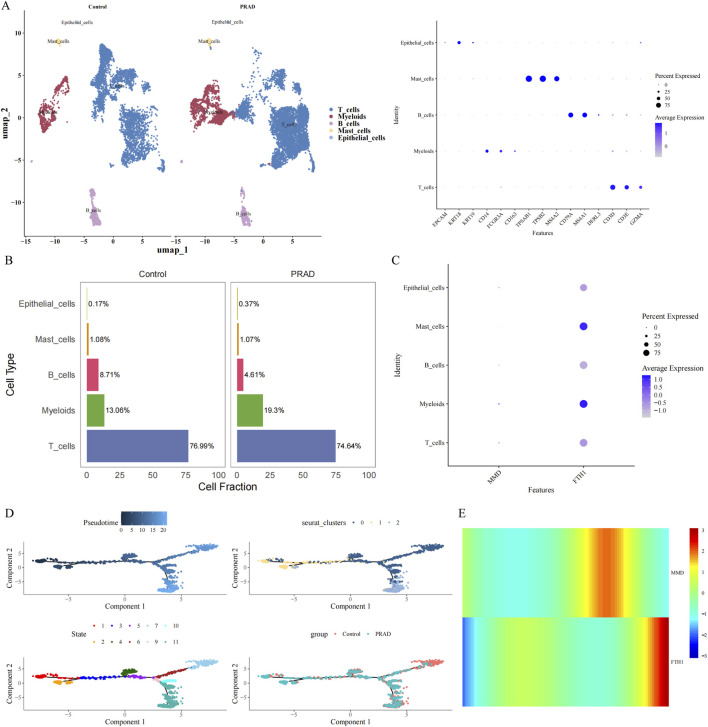
Single-cell transcriptomic profiling revealed myeloid-specific expression of prognostic genes and developmental dynamics in PRAD. **(A)** Uniform manifold approximation and projection (UMAP) plot showing five annotated major cell types based on established marker genes: epithelial cells, myeloid cells, mast cells, T cells, and B cells. **(B)** Cellular composition between control and PRAD tumor samples. **(C)** MMD and FTH1 expression across all identified cell types. **(D)** Pseudotemporal trajectory analysis of myeloid cell sub-clusters reconstructing the developmental continuum. **(E)** Expression dynamics of MMD and FTH1 along the pseudotime axis during myeloid cell development.

Analysis of the scRNA-seq data identified distinct cellular landscapes in PRAD tumor samples and controls. Following rigorous quality control and normalization, 19 distinct cell clusters were resolved (Supplementary Figure S1). Annotation based on established marker genes (Huang et al., 2023) defined five major cell types: epithelial cells, myeloid cells, mast cells, T cells, and B cells (Figure 7A). Cellular composition analysis revealed that both control and tumor samples were dominated by T cells (76.99% vs. 74.64%), while myeloid cell populations were significantly increased in PRAD tumors compared to controls (19.3% vs. 13.06%) (Figure 7B). Expression analysis across all cell types showed that both MMD and FTH1 were predominantly expressed in myeloid cells, with FTH1 exhibiting particularly prominent expression (Figure 7C). Sub-clustering of myeloid cells revealed three transcriptionally distinct subpopulations (Supplementary Figure S2). Pseudotemporal trajectory analysis reconstructed the developmental continuum of myeloid cells, with cells progressing from an early developmental state toward mature states (Figure 7D). Expression analysis along the pseudotime axis revealed distinct patterns: MMD expression was elevated during early-to-mid pseudotime but declined in late-stage cells; FTH1 displayed progressive accumulation throughout myeloid maturation, reaching peak expression in terminally differentiated states (Figure 7E). These divergent kinetics suggested that MMD might regulate early myeloid activation, while FTH1 likely governs iron homeostasis in mature myeloid cells within the TME.

### 3.8 Distinct functional characterization and cell-cell communication networks

**FIGURE 8:**
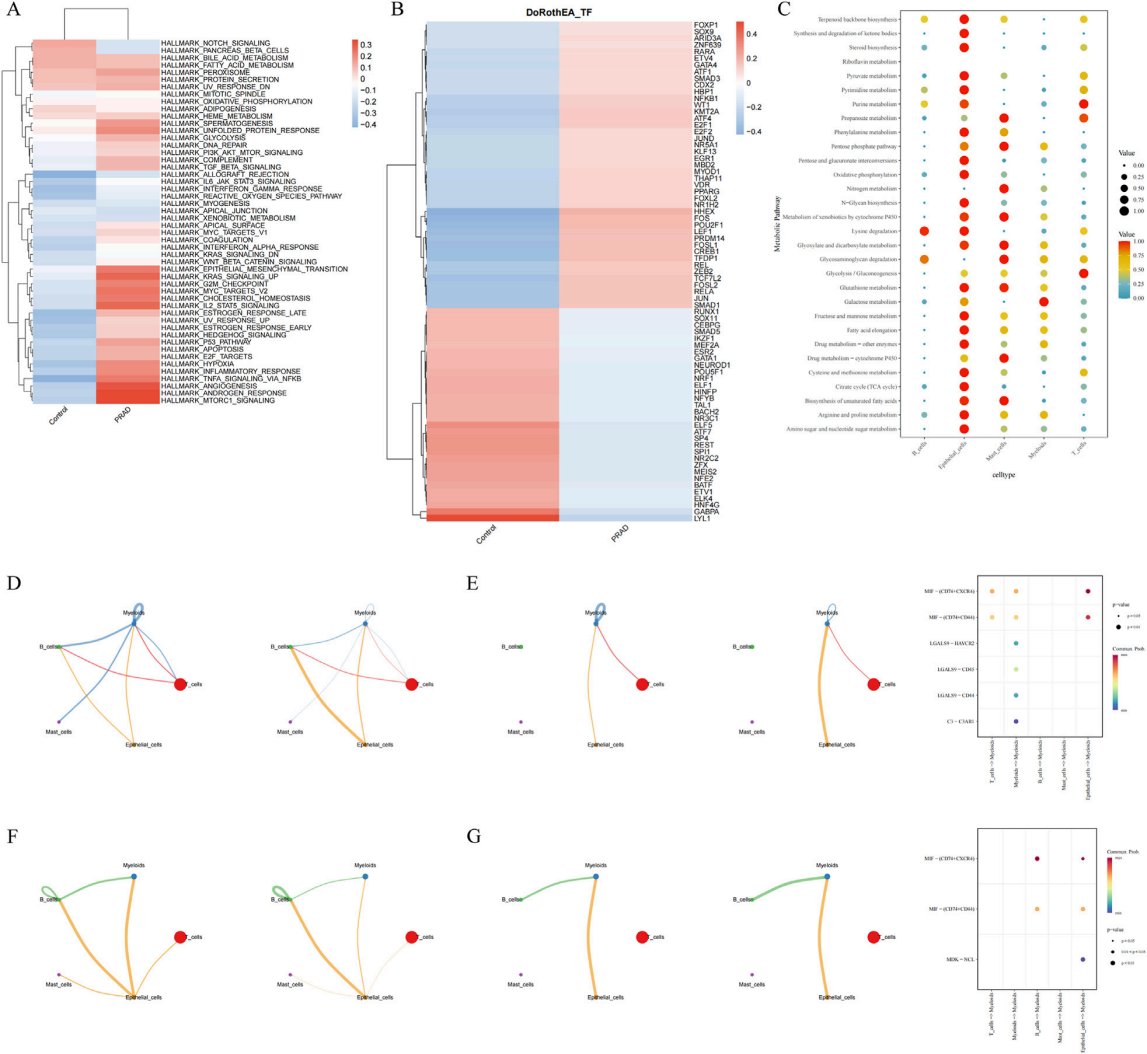
Distinct functional characterization and cell-cell communication networks in PRAD. **(A)** Heatmap of hallmark pathway enrichment scores comparing PRAD tumor samples versus control samples. **(B)** Transcription factor (TF) activity analysis comparing PRAD and control myeloid cells. **(C)** Metabolic pathway enrichment scores across major cell types. **(D)** Cell-cell communication network in control samples. **(E)** Detailed receptor-ligand interaction analysis for myeloid cells in control samples. **(F)** Cell-cell communication network in PRAD tumor samples. **(G)** Detailed receptor-ligand interaction analysis for myeloid cells in PRAD tumor samples. They are respectively net circle number and net circle weight.

Functional characterization of epithelial cells between PRAD samples and controls revealed significant differences. PRAD-derived myeloid cells exhibited significantly higher enrichment scores for hallmark gene sets, including KRAS signaling, IL2-STAT5 signaling, angiogenesis, androgen response, and MTORC1 signaling (Figure 8A). Transcription factor activity analysis displayed marked differences in transcriptional regulator activation between PRAD and control myeloid cells. Multiple TFs showed elevated activity in PRAD myeloid cells, including HHEX, FOS, POU2F1, LEF1, PRDM14, FOSL1, CREB1, and TFDP1. Conversely, GABPA and LYL1 exhibited reduced activity in PRAD samples (Figure 8B). Distinct metabolic pathway activities were observed across cell types. Myeloid cells demonstrated higher enrichment in Galactose metabolism. Epithelial cells exhibited higher enrichment scores for terpenoid backbone biosynthesis, synthesis and degradation of ketone bodies, and steroid biosynthesis (Figure 8C). Cell-cell communication analysis revealed dynamic shifts in signaling networks between PRAD tumor samples and controls. In control samples, myeloid cells engaged primarily in intensive communication with B cells (Figure 8D). Focusing on myeloid cells, they received signals from epithelial cells via MIF − (CD74+CXCR4) and MIF-(CD74^+^CD44) (Figure 8E). In PRAD tumor samples, the communication landscape was substantially altered. Myeloid cells engaged primarily in intensive communication with B cells and epithelial cells (Figure 8F). Focusing on myeloid cells, they received signals from B cells and epithelial cells via MIF − (CD74+CXCR4) (Figure 8G).

### 3.9 Potential combined targeted-immunotherapy regimens for prostate cancer treatment

**FIGURE 9:**
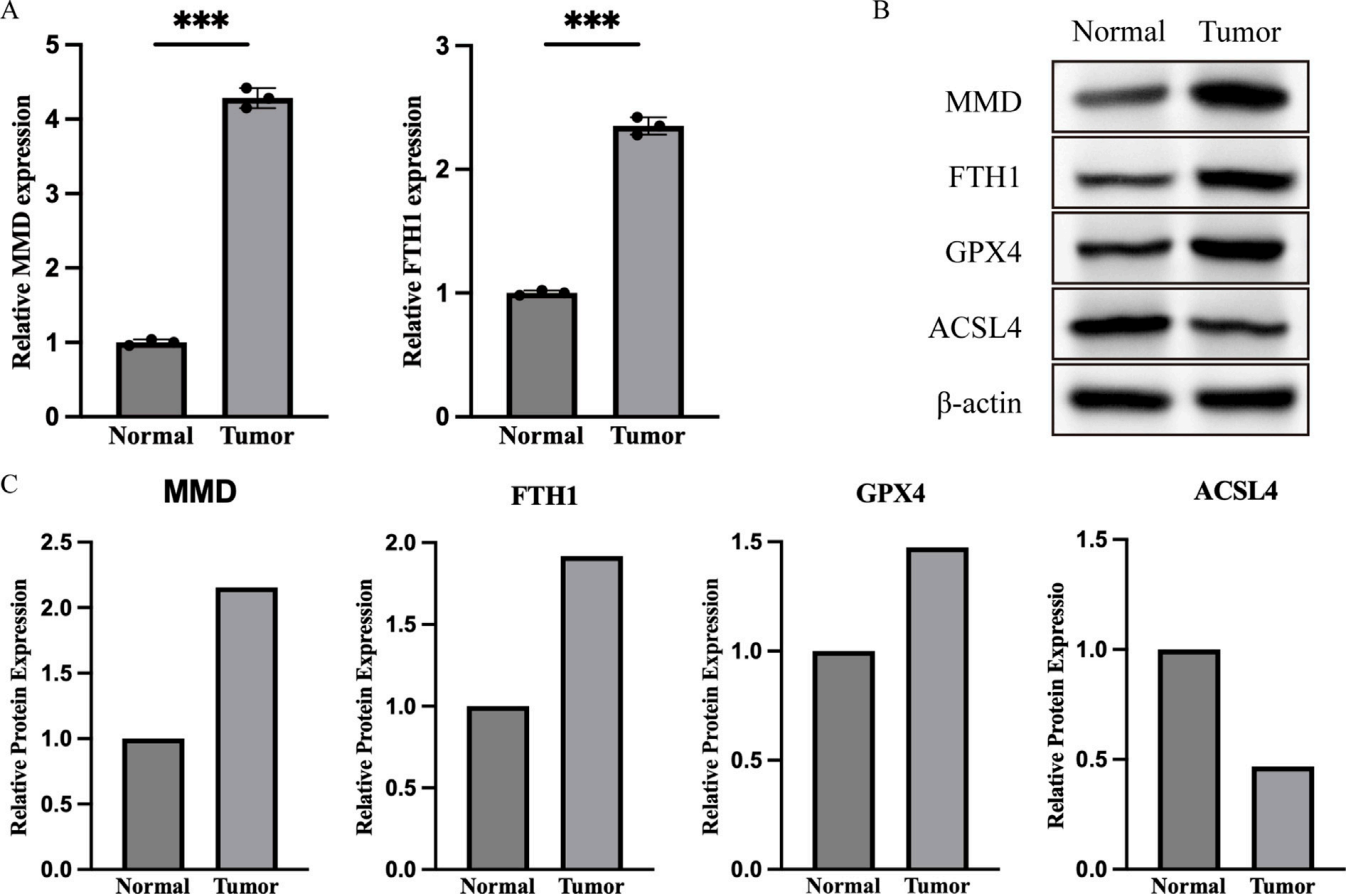
Validation of the hub genes in experimental samples. **(A)** The mRNA expression of MMD and FTH1. **(B,C)** The protrin expression of MMD and FTH1 in tissue from SCID models of prostate and C4-2 Xenograft (n = 3, ***p < 0.001).

**FIGURE 10:**
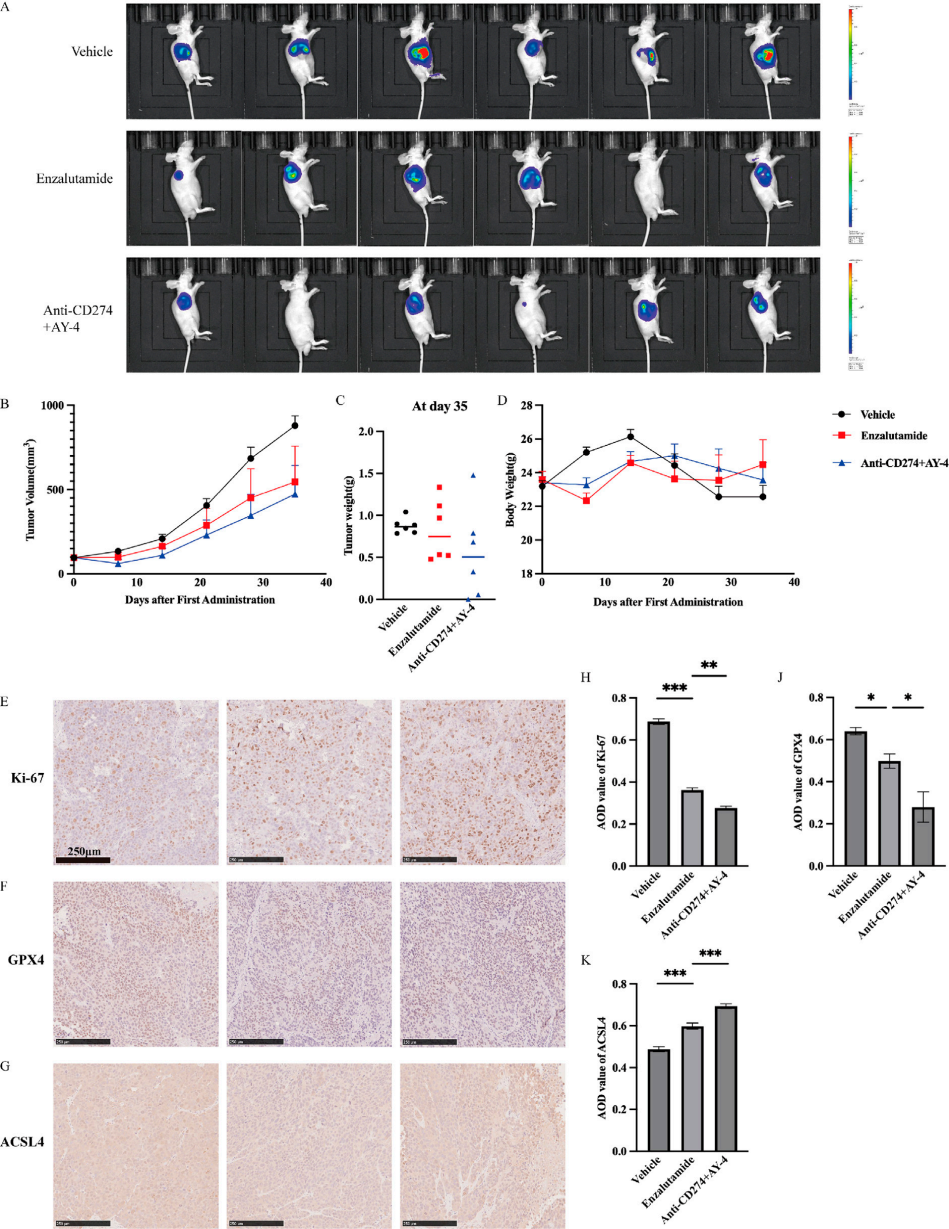
Preliminary pharmacodynamic evaluation of combined FTH1-targeted and immune-checkpoint-related treatment. **(A)** The effects of different treatment methods on tumors were shown by *in vivo* imaging (n = 6). The change in **(B)** Tumour volume, **(C)** Tumour weight, and **(D)** Body weight of each group of mice. **(E–K)** IHC staining for Ki-67, GPX4, and ACSL4 on tumor from Xenograft (n = 3).

In tissue specimens, the high expression of MMD and FTH1 in tumors was validated (Figures 9A–C). GPX4 and ACSL4 are key proteins mediating ferroptosis (Ding et al., 2023; Zhang W. et al., 2024). The pathological status of these two proteins was found to be associated with the expression of MMD and FTH1 (Figure 9C). AY-4, a potential PROTAC targeting FTH1 for degradation, demonstrated synergistic therapeutic potential when combined with anticancer drugs, and was shown to significantly increase intracellular ferrous iron levels (Ai et al., 2025). CD274 binding to its receptor PDCD1/PD-1 (programmed cell death 1) is mainly responsible for cancer cell evasion of T-cell-mediated immune surveillance (Gou et al., 2020). Subsequently, the pharmacodynamic efficacy of a combined targeted-immunotherapy regimen involving an FTH1 inhibitor and CD274 was evaluated in an animal model of advanced prostate cancer (C4-2). Castration-resistant male mice treated with enzalutamide (an AR antagonist) exhibited moderate antitumor effects. However, treatment with AY-4 combined with CD274 resulted in effective inhibition of tumor growth (Figures 10A–C). No marked changes in body weight were observed throughout any of these treatment courses (Figure 10D). Histological evidence indicated that following combination therapy, tumors showed significant downregulation of the proliferation marker Ki-67 and the ferroptosis marker GPX4, alongside significant upregulation of the ferroptosis marker ACSL4. These findings suggest the potential of a therapeutic strategy combining FTH1 inhibition-mediated lysosomal ferroptosis with immunotherapy for the treatment of prostate cancer.

## 4 Discussion

PRAD ranks among the frequently malignancies in men globally. Existing clinical prognostic indicators still have significant limitations in predicting individual long-term survival outcomes, highlighting the urgent need for novel prognostic models based on molecular characteristics to achieve precise risk stratification. Lysosomal ferroptosis-associated genes are involved in tumor progression by regulating iron homeostasis, lipid peroxidation, and tumor metabolic reprogramming, yet their prognostic value in prostate cancer has not been systematically elucidated. Through differential expression analysis and intersection with a lysosome-related ferroptosis gene set, this study ultimately identified seven CGs: CTSB, FTH1, SLC7A5, TNFAIP3, NEDD4, MMD, and MT3. GSEA enrichment analysis further displayed that these genes were commonly positively enriched in pathways linked to ribosome biogenesis, oxidative phosphorylation, and endocytosis/lysosome function, suggesting their synergistic role in driving energy metabolic reprogramming and lysosomal dysfunction in tumor cells. Notably, FTH1 and MMD showed particularly prominent enrichment in lysosomal pathways, directly linking them to the dysregulation of lysosomal iron homeostasis. Conversely, multiple genes were commonly negatively enriched in pathways such as lipid metabolism and innate immune sensing, indicating that lysosome-related ferroptosis genes may cooperatively promote prostate cancer progression by suppressing anti-tumor immune surveillance.

As core prognostic genes, FTH1 and MMD exhibit multi-dimensional pro-tumor mechanisms in PRAD and synergistically remodel the tumor immune microenvironment through shared pathways. FTH1 encodes the heavy chain subunit of ferritin, the core component of the ferritin 24-mer, which confers ferroptosis resistance to tumor cells by sequestering free iron ions and inhibiting the Fenton reaction. In the ferritinophagy pathway, NCOA4 forms liquid condensates through homotypic polymerization and multivalent interactions with FTH1, mediating the transport of ferritin particles to lysosomes for degradation via macroautophagy or ESCRT-dependent endosomal microautophagy, thereby releasing free iron to promote ferroptosis (Ohshima et al., 2022).

MMD (also known as PAQR11) is a count of the progesterone and adiponectin receptor family. It is highly induced during monocyte-to-macrophage differentiation, promotes cell survival by activating the MAPK signaling pathway, and is regulated by the transcription factor C/EBPβ (Liu and Ling, 2025). Both FTH1 and MMD were negatively enriched in the cytosolic DNA-sensing pathway, suggesting their potential synergy in suppressing innate immune surveillance and facilitating tumor immune evasion. They were also commonly negatively enriched in lipid metabolism pathways, such as linoleic acid metabolism and steroid hormone biosynthesis, indicating a potential cooperative role in promoting PRAD progression by interfering with androgen-related lipid metabolic axes. These findings collectively outline the central roles of FTH1 and MMD in regulating lysosomal iron metabolism and remodeling the tumor immune microenvironment.

The composition of the tumor immune microenvironment is a key determinant of immunotherapy response. Systematic immune infiltration analysis of the HRG and LRG in this study revealed an immunosuppression-dominated TME signature in the HRG. The proportions of activated CD4 T cells, effector memory CD8 T cells, immature B cells, MDSCs, NKT cells, and Tregs were markedly elevated in the HRG, while monocyte infiltration was markedly reduced. Although the number of effector memory CD8 T cells increased, their strong direct linked to MDSCs suggests they are not antagonistic but rather coexist synergistically within the immunosuppressive TME. MDSCs, by downregulating the expression of genes linked to PMN-MDSC recruitment and secreting immunosuppressive factors such as arginase and reactive oxygen species, directly inhibit T cell proliferation and function, serving as a critical barrier to effective anti-tumor immune responses (Chen et al., 2023). Previous studies have confirmed that combining CN133, which targets PMN-MDSCs, with anti-PD-1 therapy significantly enhances anti-tumor efficacy in soft tissue prostate cancer, highlighting the clinical translational value of MDSC depletion strategies combined with ICIs (Chen et al., 2023).

In addition to remodeling the immune microenvironment, drug sensitivity analysis further untangled differential therapeutic responses among distinct risk groups. After false discovery rate (FDR) correction, three compounds—SB505124, AZD7762, and Uprosertib—exhibited significantly different IC50 values between the high- and low-risk groups. Notably, MMD expression was positively correlated with sensitivity to SB505124 (r = 0.31, P < 0.001) and negatively correlated with sensitivity to AZD7762 (r = −0.33, P < 0.001), suggesting that MMD may serve as a potential biomarker for guiding the selection of therapeutic agents in patients with PRAD. These findings further reinforce the clinical utility of this risk model, which is applicable not only for prognostic stratification but also for predicting therapeutic response.

Through single-cell analysis, myeloid cells have been recognized as the primary cell lineage expressing prognostic genes. Myeloid cells—which include macrophages, and granulocytes—constitute a main element of the TME and drives regulating tumor progression and metastasis (van Vlerken-Ysla et al., 2023). Contrasted to adjacent normal tissues, the proportion of myeloid cells was markedly higher in PRAD tumors, suggesting that the expansion of tumor-associated myeloid cells may be an important cellular basis for the high-risk phenotype. Myeloid cell subsets with immunosuppressive properties, such as tumor-associated macrophages, tumor-associated neutrophils, and MDSCs, often proliferate extensively in cancer patients. Their accumulation is closely associated with poor prognosis and resistance to chemotherapy and ICIs (Chaib et al., 2023; Park et al., 2025). Although clinical trials targeting myeloid cell regulators on a large scale have shown limited success, targeting inhibitory myeloid cells remains a significant direction in current tumor immunotherapy research (Barry et al., 2023). FTH1 maintained high expression during late differentiation stages. The iron metabolism regulation mediated by FTH1 is closely linked to lipid peroxidation and ferroptosis inhibition, supporting the long-term immunosuppressive functional state of terminally differentiated myeloid cells by maintaining their iron homeostasis (van Vlerken-Ysla et al., 2023). First, the exploration of molecular mechanisms is insufficient. Although the expression of MMD and FTH1 was validated, and the antitumor effect of FTH1 degradation combined with PD-L1 blockade was confirmed in a xenograft model, the function of MMD in regulating prostate cancer progression and the molecular pathway through which FTH1 mediates lysosomal ferroptosis remain poorly understood. Furthermore, SCID mice lack adaptive immunity, precluding the assessment of T cell-dependent immune responses. Second, the analysis of cellular heterogeneity at the single-cell and spatial levels is lacking. The study only confirmed the specific expression of MMD and FTH1 in myeloid cells, without elucidating the spatial distribution and functional differences between high- and low-risk subtypes, thereby diminishing the predictive efficacy of subtype-specific treatment responses. Future studies could integrate single-cell and spatial transcriptomics for more in-depth analyses (Lai et al., 2025). Third, each cohort used an independent optimal cutoff value to stratify risk groups. While this approach improved stratification performance within individual cohorts, it reduced model standardization and cross-cohort generalizability. Future studies could adopt established prognostic modeling approaches, such as using a fixed cutoff value from the development set or continuous risk score modeling (Zhang Z. et al., 2022). Fourth, the model was constructed based on absolute gene expression levels, rendering it susceptible to interference from sequencing platforms and batch effects. Compared with gene pair models that are more robust to technical variations, the current model exhibits limited stability. Future efforts could optimize model generalizability by constructing gene pair features. Fifth, transcriptomic analyses can only demonstrate correlations and cannot exclude reverse causation or confounding variables. Future studies could employ Mendelian randomization, integrating GWAS and eQTL data to establish causal associations between genes and prostate cancer prognosis (Zhang C. et al., 2024). Future studies will employ a multicenter prospective cohort to validate the predictive value of this risk model in independent clinical specimens and appraises its utility in guiding clinical decision-making.

## Data Availability

The original contributions presented in the study are included in the article/Supplementary Material, further inquiries can be directed to the corresponding author.

## Supplementary material

SUPPLEMENTARY FIGURE S1 Quality control and initial clustering of scRNA-seq data. **(A)** Violin plots displaying quality metrics before filtering. **(B)** Plot of HVGs identified using the FindVariableFeatures function. The top 2,000 HVGs were selected for downstream analysis. **(C)** PCs showing the proportion of variance explained by each PC. The first 30 PCs capturing the majority of biological variation were selected for dimensionality reduction. **(D)** Based clustering of cells using the first 30 PCs, resolving 19 preliminary cell clusters before annotation.

SUPPLEMENTARY FIGURE S2 Sub-clustering of myeloid cell subpopulations. **(A)** The first 20 PCs were selected for dimensionality reduction. **(B)** Based clustering of cells using the first 20 PCs, resolving three cell clusters.

SUPPLEMENTARY TABLE S1 List of 39 lysosomal ferroptosis-related genes.

SUPPLEMENTARY TABLE S2 DEGs in TCGA-PRAD Cohort.

SUPPLEMENTARY TABLE S3 GSEA enrichment results of the candidate gene CTSB in the TCGA-PRAD cohort.

SUPPLEMENTARY TABLE S4 GSEA enrichment results of FTH1 in the TCGA-PRAD cohort.

SUPPLEMENTARY TABLE S5 GSEA enrichment results of TNFAIP3 in the TCGA-PRAD cohort.

SUPPLEMENTARY TABLE S6 GSEA enrichment results of SLC7A5 in the TCGA-PRAD cohort.

SUPPLEMENTARY TABLE S7 GSEA enrichment results of NEDD4 in the TCGA-PRAD cohort.

SUPPLEMENTARY TABLE S8 GSEA enrichment results of MMD in the TCGA-PRAD cohort.

SUPPLEMENTARY TABLE S9 GSEA enrichment results of MT3 in the TCGA-PRAD cohort.

SUPPLEMENTARY TABLE S10 GSEA of high-risk versus low-risk groups.

SUPPLEMENTARY TABLE S11 GSEA enrichment results of MMD between high- and low-risk groups.

SUPPLEMENTARY TABLE S12 GSEA enrichment results of FTH1 between high- and low-risk groups.

SUPPLEMENTARY TABLE S13 FDR-significant drugs with differential IC_50_ values between high- and low-risk groups.
